# Renal Biopsy: Use of Biomarkers as a Tool for the Diagnosis of Focal Segmental Glomerulosclerosis

**DOI:** 10.1155/2014/192836

**Published:** 2014-02-25

**Authors:** Crislaine Aparecida da Silva, Mariana Molinar Mauad Cintra, Eliângela de Castro Côbo, Marcos Vinícius da Silva, Fabiano Bichuette Custódio, Rosana Rosa Miranda Corrêa, Lúcio Roberto Castellano, Marlene Antônia dos Reis, Juliana Reis Machado

**Affiliations:** ^1^Pathology Laboratory, Nephropathology Service, Federal University of Triângulo Mineiro, 38015-150 Uberaba, MG, Brazil; ^2^Immunology Laboratory, Department of Biological Sciences, Federal University of Triângulo Mineiro, 58051-900 Uberaba, MG, Brazil; ^3^Human Immunology Research and Education Group, Technical Health School of UFPB, Federal University of Paraíba, 38025-180 João Pessoa, PB, Brazil

## Abstract

Focal segmental glomerulosclerosis (FSGS) is a glomerulopathy associated with nephrotic syndrome and podocyte injury. FSGS occurs both in children and adults and it is considered the main idiopathic nephrotic syndrome nowadays. It is extremely difficult to establish a morphological diagnosis, since some biopsies lack a considerable quantifiable number of sclerotic glomeruli, given their focal aspect and the fact that FSGS occurs in less than half of the glomeruli. Therefore, many biological molecules have been evaluated as potential markers that would enhance the diagnosis of FSGS. Some of these molecules and receptors are associated with the pathogenesis of FSGS and have potential use in diagnosis.

## 1. Introduction

Focal segmental glomerulosclerosis (FSGS) is a renal disease characterized by nephrotic syndrome that accounts for 20% of cases in children and 40% of cases in adults [1, 2]. Nearly from seven to fifteen percent of all children affected by idiopathic nephrotic syndrome are diagnosed with FSGS after renal biopsy. Without treatment, these patients might develop progressive proteinuria and a reduced glomerular filtration rate and about 30% of them might develop end-stage kidney disease in twenty years [3]. Given its morphological aspects, this glomerulopathy is defined as segmental due to the location of the lesions, and it is named focal because there are few glomeruli with sclerotic changes. FSGS is considered a podocytopathy mainly because of podocyte injury and the subsequent loss of such an important structure of the glomerular filtration barrier. Proteinuria is a defining clinical characteristic of focal segmental glomerulosclerosis [1].

Although some genetic mutations in podocyte genes have been linked to the occurrence of FSGS in humans, many patients develop this clinical entity without any genetic disorders, whereas other patients have proteinuria after some hours or even days after kidney transplantation. These clinical manifestations have shed some light on the hypothesis that a possible circulating factor would be involved in FSGS [4]. The concept of FSGS permeability factor is highly supported by recurrent syndrome after transplantation [5], proteinuria response to the plasmapheresis therapy [6], immunoadsorption [7], and transient nephrotic syndrome in a newborn whose mother had the disease [8].

Some studies have shown that patients with recurrent FSGS have some permeability factors on plasma that would be responsible for injuring the glomerular filtration barrier, thus causing proteinuria [9, 10]. Among these factors, some have been receiving considerable attention: CCL1, SuPAR, and hemopexin, although the last has been better correlated to minimal change disease (MCD) [11–13].

The search for markers that could be useful as diagnostic tools for FSGS is extremely important, since few renal biopsies of FSGS patients have a considerable number of injured glomeruli to determine the actual diagnosis of the disease. In order to facilitate diagnosis, researchers have speculated that some markers, such as components of the glomerular basement membrane, cytokines, receptors, and matrix components, as well as their soluble forms and oxidative stress markers, could be potential future diagnostic tools for FSGS (summarized in Table 1).

## 2. SuPAR

SuPAR is the soluble form of the urokinase-type plasminogen activator receptor (uPAR), and it is expressed in several immunologically active cells. Increased serum levels are indicative of immune system activation in inflammatory conditions and infections [14], such as HIV, tuberculosis, malaria, sepsis, rheumatoid arthritis [15], and FSGS [16].

The form associated with plasma membrane (uPAR) is an important regulator of plasminogen activation and a major component of homeostasis expressed on the surface of a wide variety of cells [17, 18]. The biological activity of SuPAR is not fully known, so it may be either an active molecule or simply the result of an increased release of uPAR on the cell surface. Nonetheless, uPAR interacts with both uPA and vitronectin, and the latter interacts with integrins in order to regulate cell motility [19, 20].

This receptor has also been shown to orchestrate signaling pathways by binding to other transmembrane receptors, including integrins [21, 22]. The urokinase receptor is composed of a glycosylphosphatidylinositol- (GPI-) anchored protein with three domains that confer solubility to it when detached from the membrane by cleavage of GPI anchors [21].

SuPAR is expressed at low levels under physiological conditions in human blood and it has a known role as a circulating protein involved in neutrophil trafficking and stem cell mobilization [21, 22]. Its ability of binding to integrins is essential for activating them [27]. Integrins, in turn, might determine podocyte motility during the early manifestations of FSGS in native and posttransplant kidneys [53].

Regardless of the stimuli (via local podocyte or systemic), pathological integrin signaling in podocytes might be considered a key event in the induction of glomerular diseases associated with proteinuria [16]. However, other permeability factors may be involved, either alone or in conjunction with SuPAR, in some nephropathies such as that observed in approximately 70% of patients with membranous nephropathy who have anti-PLA2R antibodies [54].

The SuPAR receptor was evaluated as a cause of primary FSGS in different experimental models of disease, showing that SuPAR may be deposited in the glomeruli and/or bound to podocyte integrins, hence activating them to cause loss of cell function as well as detectable proteinuria [27]. A recent study identified SuPAR in plasma samples of recurrent FSGS patients. Downstream signaling pathway activation of uPAR receptor expressed in podocyte membranes causes the loss of function of these receptors and detectable proteinuria [16]. Moreover, high SuPAR serum concentrations were found in patients with primary FSGS, but not in those affected by other glomerulopathies, such as minimal change disease (MCD), membranous nephropathy, and preeclampsia [27].

Another paper,  however, showed that SuPAR was elevated during primary FSGS while comparing the plasma levels of this soluble factor among patients with GESF, which may limit its usefulness to differentiate forms of this disease [23]. Moreover, this same work has shown that SuPAR levels would be also detected at high levels on patients with more severe kidney lesions as tubular atrophy, interstitial fibrosis, and inflammation cells infiltrate [23]. On the other hand, Wei et al. (2012) [24] could not correlate elevated levels of SuPAR with elevated C-reactive protein levels as an inflammatory marker during comparison made between two groups of GESF patients from distinct regions. This work also demonstrated that primary FSGS patients present high SuPAR levels independently of being adults or children [24]. These results diverge from that observed by Bock et al. (2013) [26] who demonstrated that SuPAR levels would not contribute to primary FSGS diagnosis among pediatric patients.

Despite all these evidences in the last years, it is not yet clear why FSGS patients have high circulating SuPAR levels. It has been speculated that there might be a connection between mutations in podocyte genes [24, 55] and an increase in some circulating permeability factors, even though these events occur by accident. On the other hand, abnormalities in podocytes might progress to a more aggressive lesion that would be responsible for an increased feedback loop between circulating molecules, thus leading to recurrent or even more severe forms of renal failure [55].

In cases of renal failure, higher serum and urine levels of proteins and more precisely of albumin seem to have a direct correlation with SuPAR levels in all kidney transplant candidates comparing to healthy controls. Nevertheless, these higher SuPAR levels seemed to not present any specificity to FSGS, albeit elevated SuPAR levels were detected in the urine of transplanted patients with recurrent FSGS [25].

Taking all these data together, we believe that the measurement of SuPAR levels might be useful diagnostic tool for primary FSGS, thus indicating its implementation in a clinical routine panel together with other biomarkers in pretransplanted patients.

## 3. CLC-1

CLC, also referred to as CLCF1 (Cardiotrophin-like cytokine factor 1), NNT-1, or BSF-3 (Neurotrophin-1/B-cell-stimulating factor-3, also known as cardiotrophin-like cytokine) are cytokines identified based on their homology with the IL-6 family [56, 57].

CLC is expressed in the immune system and its cDNAs could be cloned from T cells [58, 59]. It is an important cytokine implicated in the development of the central nervous system [57], is involved in motor neuron development *in vitro*, and its transcripts are found in the skeletal muscle during massive motor neuron cell death [60, 61].

The CLC-1 was identified in the active fraction by galactose affinity chromatography, and it was isolated as a protein that mediates albumin permeability (Palb). The Palb assay values vary from 0, in normal glomeruli, to 1.0 in tissues with a maximum lesion diameter. Nearly 30% of patients with FSGS have a Palb value of 0.5 [13].

Recently, CLC-1 has been described as a soluble factor related to recurrent FSGS cases. Studies have shown that CLC-1 circulating levels in patients with recurrent FSGS may be up to 100 times higher than in normal subjects; however, its genesis still needs clarification [4]. Until now, it is known that CLC owns important neutrophilic activity, with interesting immunomodulatory effects after its interaction with the soluble receptor CLF [56, 62]. This receptor for CLC-1 seems to be intimately related to other matrix proteins, modulating the intensity of type III collagen deposition in the liver [63].

In the kidney, CLC-1 seems to increase the glomerular permeability, whereas decreasing the expression of nephrin [4]. Further studies are needed to elucidate the role of CLC-1 in FSGS pathogenesis.

## 4. TGF-***β***

Transforming growth factor-beta (TGF-*β*) is a multifunctional protein that controls the proliferation, differentiation, and other functions of many cell types. Many cells synthesize TGF-*β* and have specific receptors for it; they also regulate, positively and negatively, many other growth factors. TGF-*β* plays an important role in the bone remodeling process as a potent stimulator of osteoblastic bone formation, leading to chemotaxis, proliferation, and differentiation in compromised osteoblasts. It is highly expressed in bones and abundantly expressed in articular cartilage and chondrocytes, and it is increased in osteoarthritis as well. A mutation in TGF-*β* is associated with skeletal diseases such as Camurati-Engelmann disease (CED), also known as progressive diaphyseal dysplasia 1 (PDD1) [64–69].

The pathogenesis of glomerular sclerosis observed in FSGS patients may be caused by an increase in glomerular profibrotic cytokines, such as IL-13, IL-4, or TGF-*β* [70–73]. Increased levels of TGF-*β* may be linked to the presence of intrinsic sclerosis pathways that stimulate cytokine production, such as high glomerular intracapillary pressure [74, 75], glomerular hypertrophy [76], and excessive protein loss through glomerular capillaries [77].

TGF-*β* is a key regulator of the extracellular matrix surrounding renal cells. Its expression by podocytes might contribute to glomerular basement membrane thickness, to mesangial matrix expansion, and to apoptosis [78]. The production of TGF-*β* is controlled by cell-cycle regulatory proteins. The activation of specific cyclin-dependent kinases (CDK) promotes cell proliferation. The cyclin-CDK complex is negatively regulated by CDK inhibitors p21, p27, and p57. Podocyte injury induces the loss of function of p27 and p57, resulting in uncontrolled proliferation of podocytes [31].

Furthermore, Smad2 and Smad3 are molecules that mediate cell growth and that are activated by TGF-*β* receptors. Phosphorylated Smad2/Smad3 then form complexes with Smad4 and move into the nucleus to transduce signals to target genes [79]. Increased expression of TGF-*β*, of TGF-*β* receptor type II, and of phosphorylated Smad2/3 proteins is observed in podocytes of FSGS patients [30]. Nevertheless, TGF-*β* itself may induce the expression of Smad7, which inhibits Smad2/3 phosphorylation in a negative feedback loop [80]. Smad7 is highly expressed in patients with clinical signs of podocyte injury. *In vitro* culture of podocytes in the presence of TGF-*β* showed that both Smad7 and TGF-SS1 may be associated with cell apoptosis, suggesting that Smad7 has a role in progressive podocyte loss [32].

Some studies have demonstrated that increased renal expression of TGF-*β*1 may be observed in children with FSGS in contrast with patients with LM. This observation suggests that the transcription of the TGF-*β* gene in kidneys might predict the development of kidney failure in FSGS [29].

The increased production of TGF-*β* might induce the expression of the integrin-linked kinase (ILK), a protein which is related to the pathogenesis of many nephropathies associated with proteinuria. The upregulation of ILK in podocytes might determine the occurrence of epithelial-mesenchymal transition (EMT) in these cells by inducing helix-loop-helix transcription factors [81].

## 5. Malondialdehyde

Malondialdehyde (MDA) is a natural product formed in all mammalian cells as an end-product of lipid peroxidation. MDA is a highly reactive three-carbon dialdehyde and it is produced as a peroxidation product of polyunsaturated fatty acids, including the metabolism of arachidonic acid. MDA immediately binds to several functional groups of molecules, including proteins, lipoproteins, and DNA. It also reacts with DNA to form adducts to deoxyguanosine and deoxyadenosine. The major adduct to DNA is a pyrimidopurinone called M1G, which appears to be a major endogenous DNA adduct in human beings, and which may significantly lead to cancer associated with lifestyle and dietary factors. MDA-modified proteins may show altered physico chemical behavior and antigenicity. Because MDA is toxic, it has been implicated in aging, mutagenesis, carcinogenesis, diabetic nephropathy, and radiation damage. Increased expression of MDA has been observed in the brains of Alzheimer's patients [82–88].

Malondialdehyde (MDA) is an important biomarker of oxidative stress. In general, lipid peroxidation products such as malondialdehyde (MDA, C3H4O2) may be used as indicators of the action of free radicals in the body. MDA has cytotoxic and genotoxic activities, as well as increased levels in some diseases associated with oxidative stress; in addition, MDA quantification in biological samples accounts for an important oxidative stress parameter [33].

Biological membranes have high levels of polyunsaturated acids that make them susceptible to attack by reactive oxygen species (ROS) [89, 90]. Antioxidants can neutralize ROS, but, in some situations in which the balance has been lost, the phenomenon of oxidative stress arises. Even though all cellular components are susceptible to the action of ROS in the oxidative burst, the membrane is one of the most affected targets, especially after lipid peroxidation, which alters its structure and permeability [91].

Given the challenging aspect of direct measurement of free radicals in tissues, lipid peroxidation is often used as a predictive index of ROS-mediated injuries. Malondialdehyde is a marker of oxidative stress-induced lipid peroxidation that has been regarded as the best marker for several acute and chronic kidney diseases [34, 35].

Binder and colleagues demonstrated that Mpv17 gene-inactivated mice presented glomerular overproduction of oxygen radicals, which can be used as a model of FSGS in humans [38]. Higher levels of malondialdehyde were observed in urine and serum samples, as well as its glomerular overexpression in patients with FSGS in comparison with minimal change disease (MCD) [36, 37]. Therefore, tissular expression of oxidative stress markers should be regarded as an important tool for the diagnosis of renal injury.

## 6. Dystroglycan

Dystroglycans are essential elements of the neuromuscular junction (NMJ). The dystroglycan gene is expressed as a precursor protein that is posttranslationally cleaved inside a 156 kDa extracellular peripheral membrane protein called alpha-dystroglycan and a 43 kDa transmembrane protein, beta-dystroglycan. The latter is a PPxY motif-containing protein that binds to a WW domain-containing protein, such as utrophin and dystrophin. Phosphorylation at tyrosine 892 within the PPxY motif can regulate c-Src interactions with beta-dystroglycan, as well as inhibiting interaction with WW domains. Beta-dystroglycan is normally localized to the skeletal muscle cell plasma membrane; however, phosphorylation at Tyr-892 leads to localization of beta-dystroglycan to endosomal compartments along with c-Src. Therefore, phosphorylation at Tyr-892 may have a central role in altering the localization of beta-dystroglycan during NMJ formation [92–98].

In kidneys, *α*-/*β*-dystroglycans (DG) are matrix receptors that mechanistically serve as anchors to the glomerular filtration membrane [41]. The *β*-chain contains the polyanionic binding site for the cationic laminin globular domain common to several matrix proteins, such as agrin, perlecan, proteoglycans, and laminin itself [99, 100]. The *β*-chain, on the other hand, binds noncovalently to the DG complex in the actin cytoskeleton [101, 102]. Regele et al. (2000) [39] observed that MCD patients had decreased *α*,*β*-DG levels, whereas normal levels were found in patients with FSGS. On the other hand, despite the lower levels of 3-DG in MCD, they were increased in FSGS. Furthermore, a study evaluating the glomerular expression of DG in renal biopsies showed an increased expression of DG in FSGS cases compared with MDC cases [40].

## 7. TRP6

The transient receptor potential cation channel 6 (*trpc6*) is a gene located in the long arm of chromosome 11 (11q22.1) encoding a protein of the same name [42, 43]. It is considered to be a receptor-activated nonselective calcium permanent cation channel [103]. The TRPC channels comprise a family of nonselective Ca^2+^ permeable cation channels that are widely expressed in vertebrate tissues [42, 43]. It is believed that members of the TRPC family are activated in response to signal transduction pathways involved in phospholipase C stimulation [104]. These channels have been associated with many biological functions, such as cell growth, ion homeostasis, and Ca^2+^ influx-dependent mechanical functions [105].

The TRPC6 channel is an important component of the glomerular basement membrane colocalized with CD2AP, nephrin, and podocin in podocytes [106]. It coimmunoprecipitates with dynamin [107], which plays an important role in membrane protein trafficking and cytoskeleton regulation. Functional interactions between channel proteins and podocins may help to determine the integrity of podocyte filtration functions in order to detect mechanical stimuli and to activate Ca^2+^ signaling cascades that can alter cytoskeleton dynamics [108]. TRPC6 is expressed mainly in the placenta, lungs, spleen, ovaries, and small intestine, as well as in podocytes; hence, it is a component of the glomerular slit diaphragm. Moreover, it seems to be involved in the onset of focal segmental glomerulosclerosis and focal segmental glomerulosclerosis 2 (FSGS2) [103].

The importance of TRPC6 channels in podocyte function was only suggested after the discovery that mutations in the *trpc6* gene could cause inherited glomerular diseases such as FSGS [42, 43]. Furthermore, it is hypothesized that TRPC6 mutations could alter the intracellular milieu, which, as a consequence, would induce an irreversible cell state. These observations suggest that the glomerular phenotype is the consequence of a unique role of TRPC6 in the organization of the filtration apparatus or a susceptibility of this structure to the dynamic regulation of the cytoskeleton, since patients with FSGS presenting *trpc6* mutations do not usually have any other pathological phenotypes [109, 110].

## 8. MicroRNAs (miR-192 and miR-205)

MicroRNAs (miRNAs) seem to play physiological roles in development and in pathological processes. Several miRNAs can be found in the extracellular fluids, such as plasma, serum [111, 112], and urine [113], as well as tissues and cells. MicroRNAs are relatively stable, which make them ideal biomarkers for disease. Even though many miRNAs are highly expressed in several tissues, some miRNAs are organ specific [114]. Specifically, the miR-192 and miR-205 are mainly expressed in the renal cortex and are extremely relevant to renal cell biology. These miRNAs are more abundant in the kidney than in any other organ [45], and their levels are higher in the serum from patients with focal segmental glomerulosclerosis (FSGS) in relation to the serum from patients with minimal change disease (MCD), and they were also regarded as biomarkers in the differentiation between FSGS and MCD [44].

## 9. Metalloproteinases (MMP-2 and MMP-9) and TIMPs 

Metalloproteinases are a family of zinc-dependent endopeptidases that play important roles, such as segregation of the extracellular matrix, tissue remodeling, and fibrosis in several organs, including the kidney [115, 116]. More than 28 metalloproteinases have been described to date, and they are classified into different subgroups (collagenases, matrilysin, and stromelysin) according to their structure and function [117]. Metalloproteinase-2 (MMP-2) and metalloproteinase-9 (MMP-9) are members of the gelatinase family and were demonstrated in animal models and *in vitro* systems as important mechanisms of fibrosis in progressive chronic kidney disease [115, 118]. MMP-2 is normally expressed in mesangial cells, and it shows increased expression in these cells, as well as in podocytes during inflammatory states [46]. Although MMP-9 is not expressed in the glomerulus under normal conditions, it is expressed during pathological conditions [48]. Both MMP-2 and MMP-9 are highly expressed in the tubular compartment in animal models with chronic kidney disease, and they were also found in the serum of patients with chronic kidney disease [28, 46, 47, 119].

According to Korzeniecka-Kozerska et al. (2013) [120], matrix metalloproteinase-9/neutrophil gelatinase-associated lipocalin (MMP-9/NGAL) is a better marker of focal glomerulosclerosis. In hypertensive rats with glomerulosclerosis, MMP-2 is increased in the glomerulus, but MMP-9 is not [48, 121]. In humans, MMP-2 has been described as an important intermediary in tubulointerstitial fibrosis in diabetic nephropathy [118]. MMP-9, but not MMP-2, has been found in glomerular lesions in several human kidney diseases, including nephritis in Henoch-Schönlein Purpura, IgA nephropathy, and poststreptococcal glomerulonephritis [122]. The urine of patients with FSGS has prematurely increased levels of MMPs and tissue inhibitors of metalloproteinases (TIMPs) in comparison with individuals in control groups and patients with steroid-sensitive nephrotic syndrome [123]. Catania et al. observed the same data in their study, in which they found increased levels of NGAL and of urinary MMP-9 in patients with focal segmental glomerulosclerosis in contrast with the control group. MMP-9/NGAL ratio is considered an important marker of the differentiation of minimal change disease and focal segmental glomerulosclerosis in children with nephrotic syndrome [120].

## 10. Human Neutrophil Gelatinase-Associated Lipocalin (NGAL)

Also referred to as Lipocalin-2, Siderocalin, Uterocalin, and 24*p*3 [124], neutrophil gelatinase-associated lipocalin is highly accumulated in human kidney cortical tubules and in the blood and urine after nephrotoxic and ischemic injuries [49, 50]. NGAL is significantly increased in patients with stable, but nonprogressive, renal failure [51, 125]. Urine and plasma NGAL levels increase proportionally to severity and duration of renal injury and rapidly decrease with attenuation of renal injury [126]. In glomerular diseases, analysis of isoenzymes of NGAL demonstrates that there is an increased urinary excretion of this enzyme because of an increase in the release by renal tubular cells, and not because of the increased filtration through the damaged glomerular capillary wall [127]. Urinary NGAL can be used as an acute kidney injury marker; thus, it is a marker of disease progression in children with FSGS, and it may represent disease progression in relation to its glomerular filtration rate [52].

Despite the lack of definitive studies about the role played by this molecule in the pathogenesis of kidney diseases, this seems to be a promising marker of chronic kidney diseases, being useful for disease screening or prognosis.

## 11. Conclusion

It is extremely important to understand the role played by each of the revised molecules in FSGS pathogenesis. The establishment of a complete panel of biomarkers for FSGS diagnosis is highly desirable. We believe that components of the glomerular basal membrane and the matrix, together with specific cytokines and receptors, their soluble isoforms, and oxidative stress markers, are the best candidates chosen by nephropathology services in order to provide FSGS diagnosis.

## Figures and Tables

**Table 1:** The potential use of different biomarkers on FSGS diagnosis.

Biomarker	Species	Activity	Reference
*Soluble urokinase-type plasminogen* *activator receptor *(SuPAR)	Human	Elevated levels in the plasma of primary FSGS patients and in plasma and urine of transplanted patients with recurrent FSGS	[23–25]
		Still inconclusive for primary FSGS diagnosis in children	[26]
	Mice	Causes foot process effacement, proteinuria, and FSGS-like glomerulopathy by direct activation of podocyte integrins	[16, 27]

*Cardiotrophin-like cytokine* *factor 1 *(CLC-1 )	Human	Elevated plasma levels in patients with recurrent FSGS, mimicking the effects of FSGS plasma on Palb. Anti-CLC-1- specific monoclonal antibody blocks the Palb effect of active FSGS sera	[4, 13]

*Transforming growth factor-beta* (TGF-*β*)	Human	Circulating plasma levels correlated with other biomarkers in patients with diverse glomerulopathies	[28]
		Increased renal expression in children with FSGS	[29]
		Increased expression of the protein, its receptor, and associated signalling proteins in podocytes of FSGS patients	[30]
		In situ expression is associated with changes in extracellular matrix and podocyte apoptosis	[31]
	Mice	Induces podocyte apoptosis	[32]

Malondialdehyde	Human	Potent marker of oxidative stress-induced lipid peroxidation used in several acute and chronic kidney diseases in adults and children	[33–35]
		Elevated urine and serum levels associated with glomerular overexpression in patients with FSGS	[36, 37]
	Mice	Associated with reactive oxygen species overproduction in animal glomeruli mimicking human FSGS	[38]

Dystroglycan	Human	Differential expression between MCD and FSGS patients	[39]
		Increased expression in FSGS compared to MDC renal biopsies	[40]
	Rat	Serves as matrix anchor to the glomerular filtration membrane	[41]

*Transient receptor potential cation* *channel 6 *(TRP6)	Human	Fundamental component of the glomerular basement membrane. Gene mutations cause inherited glomerular diseases such as FSGS	[42, 43]

MicroRNAs (miR-192 and miR-205)	Human	Higher serum levels in primary FSGS than MCD patients. They correlate with the degree of interstitial fibrosis in FSGS	[44]
	Rat	They are mainly expressed in the renal cortex	[45]

Metalloproteinases (MMP-2 and MMP-9) and *Tissue inhibitors of metalloproteinases* (TIMPs)	Human	Elevated plasma levels associated with kidney allograft survival	[46]
		Circulating plasma levels correlated with other biomarkers in patients with diverse glomerulopathies	[28]
	Mice	Decreased expression in the kidneys of mice presenting glomerulonephritis	[47]
	Rat	Reduced expression was associated with fibronectin deposition in FSGS injured glomeruli	[48]

*Human neutrophil gelatinase-associated* *lipocalin *(NGAL)	Human	Increase in urine and kidney cortical tubules of patients with acute renal failure	[49–51]
		Elevated levels in the urine from children with FSGS, presenting positive correlation with urinary protein excretion but negative correlation with estimated creatinine clearance at disease diagnosis	[52]
